# *Dita.te*—A Dictation Assessment Instrument with Automatic Analysis

**DOI:** 10.3390/children12060774

**Published:** 2025-06-14

**Authors:** Daniela Saraiva, Ana Margarida Ramalho, Ana Rita Valente, Cláudia Rocha, Marisa Lousada

**Affiliations:** 1 Psilexis, 1050-083 Lisbon, Portugal; danielasaraiva@psilexis.pt (D.S.); claudiarocha@psilexis.pt (C.R.); 2 Escola Superior de Saúde do Alcoitão/Centro de Linguística da Universidade de Lisboa, Faculdade de Letras, Universidade de Lisboa, 1600-214 Lisbon, Portugal; ana.ramalho@essa.scml.pt; 3 School of Health Sciences, IPLeiria and IEETA/LASI, University of Aveiro, 3810-193 Aveiro, Portugal; 4 RISE-Health and School of Health Sciences, University of Aveiro, 3810-193 Aveiro, Portugal; marisalousada@ua.pt

**Keywords:** dictation assessment instrument, automatic analysis, reliability

## Abstract

**Background/Objectives:** To date, there are no validated tools that assess children’s performance in connected text dictation tasks in European Portuguese using automated analysis. International studies were identified, but these primarily involved word dictation tasks and did not use automatic scoring tools. The present study aims to assess the reliability of the *Dita.te* (internal consistency and inter-rater reliability), a written assessment test based on a dictation task with automatic spreadsheet analysis, and establish normative data for text dictation tasks for children from 3rd to 6th grade. **Methods**: This study included 315 European Portuguese-speaking children from the 3rd to 6th grades. The *Dita.te* tool was used to assess orthographic errors based on phonological, morphological, and prosodic criteria. Descriptive statistics, percentiles, the inter-rater reliability and internal consistency were analyzed. Non-parametric tests compared performance by gender and school year due to a non-normal data distribution. **Results**: The *Dita.te* had excellent internal consistency (α = 0.929). The correlation between items scored highly (Intraclass Correlation Coefficient = 0.925). The number of errors decreased as the school year progressed, with errors affecting the syllable nucleus being the most frequent across all school years. These were followed by orthographic substitution errors, with grapheme omission being the most prevalent. **Conclusions**: Our findings suggest that orthographic competence is mostly stable before the 3rd grade, and the mismatches found in children with typical development show residual error in their orthographic performance.

## 1. Introduction

Writing assessment is an essential component of literacy assessment, providing crucial insights into an individual’s language skills, cognitive processes, and developmental status. This paper will focus on the validation of a dictation-based assessment tool with automated scoring.

Dictation provides precise control over linguistic input, allowing researchers and clinicians to systematically manipulate variables such as word frequency, phonological and morphological complexity, and orthographic regularity [1]. This controlled environment enables a detailed analysis of specific orthographic patterns and potential error sources.

Also, dictation tasks facilitate cross-linguistic comparisons, as they can be standardized across languages while accounting for language-specific features [2]. This comparability has proven valuable in identifying universal versus language-specific aspects of writing development and disorders [3].

However, most proposals aim to establish rule patterns that seek to understand children’s linguistic functioning through linguistics-based analysis models. Recently, tools and models that integrate phonological, orthographic, and morphological analysis have been tested to assess orthographic coding [4,5].

The analysis of writing errors has been studied from different perspectives in the case of European Portuguese e.g., [6,7,8]. Several studies, especially those carried out in the early stages of reading, have focused on errors by analyzing the reconstruction strategies used by children until they reach a certain level of maturity e.g., [9,10]. Other studies have examined errors in older children [11] and in those with disabilities, namely, those with written language disorders like dyslexia [6].

The existence of validated instruments is a pressing need when attempting to distinguish typical from atypical behavior. Fortunately, in recent years, recommendations for the development of assessment instruments for use in clinical contexts e.g., [12,13] have emphasized the importance of including aspects of content validity (construct and content validity) and reliability in the early stages of the development of these instruments.

Most of the theoretical proposals try to establish patterns of rules that help understand the children’s linguistic functioning. The Triple Word Form Theory [5,11] conceptualizes spelling development involving three interconnected representations: phonological, orthographic, and morphological. Analysis frameworks based on this theory examine how these representations interact during spelling tasks, providing insights into both typical development and writing disorders. This theory is important for understanding the development of literacy skills in children and the process of producing written words in dictation tasks e.g., [7,11].

Since Portuguese is a semi-transparent language in terms of the relationship between sounds and letters [14], most of the studies and classifications used have considered and explored the relationship between phonology and orthography [6,7,15].

For European Portuguese, no validated instrument has been constructed in the light of the triple word form model, which includes the control of phonological, orthographic, and morphological variables in a text dictation task. To the best of our knowledge, no previous tools using text dictation with automatic analysis have been created. There are dictation assessment instruments for other languages, but usually they use single-word dictation, not text dictation (e.g., [16]). Most assessment tools focus either on phonological analysis or orthographic analysis. Thus, in clinical settings, the most commonly used instruments are word dictation tasks, which allow a quicker assessment. However, in educational contexts, teachers often rely on error analysis based on text dictation tasks. Through the use of a text dictation task, which is common for children, we can perform a detailed analysis of writing.

Therefore, the development of an instrument that allows speech and language therapists to relatively quickly assess the different linguistic components involved in writing, and at the same time identify or rule out potential alterations, underlines the importance of developing such an instrument. Text dictation activates and evaluates a broader range of linguistic and cognitive abilities, providing deeper insight into a learner’s overall language competence.

**Figure 1:**
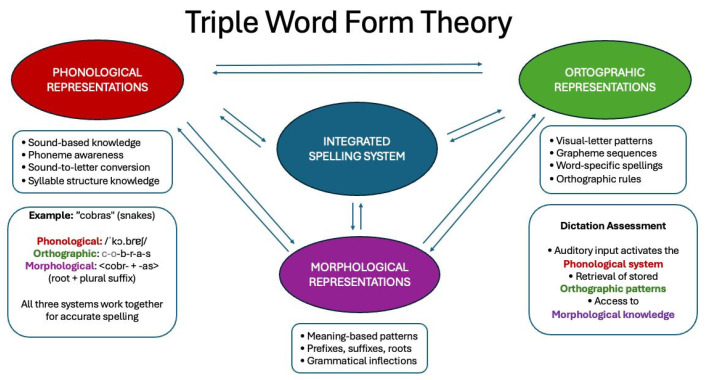
Triple Word Form Theory scheme adapted to *Dita.te* analysis.

Speech and language pathologists are also increasingly recognizing the importance of organization and time management in their clinical practice, which has led to technology being identified as a response to the time constraints associated with assessment [17,18]. As noted by [17], the significant reduction in time required for computerized analysis compared to manual analysis helps to streamline and optimize the procedures involved in the processing and analysis of assessment data. As a result, clinicians will increasingly choose tools that enable automated analysis over traditional assessment data processing and analysis methods [17]. The *Dita.te* instrument represents a significant advancement in writing assessment for European Portuguese, using a dictation task of a text as its primary methodological approach. This practical and theoretical framework provides a comprehensive review of contemporary writing assessment practices, highlights the advantages of dictation as a data collection method, and explores relevant phonological, morphological, and orthographic analysis frameworks that support the *Dita.te* assessment tool. In the dictation of a text, the children must hear a word or sentence and write it accurately. This involves the coordination of all three components of the Triple Word Form Theory, which is particularly relevant for the identification of the specific component in which a student may be experiencing difficulty. Specifically, the phonological component was considered through the analysis of phonological errors (e.g., segmental substitution errors by manner, place and voicing; omissions, insertions, substitutions and inversions that affect the syllabic structure) and the orthographic component (e.g., grapheme omissions or substitutions that occurs due to the non consolidation of orthographical rules, such as omission of the grapheme <h> that do not have a sound correspondence). Additionally, the teacher could qualitatively analyze the errors by grammatical class, such as errors with clitic pronouns and word boundary errors (morphological component) (see Figure 1).

After analyzing the difficult component, the teacher can emphasize phonics and sound–letter correspondence for struggling spellers; provide rules for orthographic development; and introduce morphemes and word families to develop vocabulary and aid in the writing of complex words.

It is particularly important to develop an instrument for European Portuguese, considering the differences between the two varieties of Portuguese (Brazilian and European). In particular, in European Portuguese, the vowel system representation is more complex due to the use of unstressed vowels, which leads to several written errors (e.g., in Portuguese, the vowel <e> may correspond to the phonetic realizations [e], [ɛ], or [i], depending on its phonological environment).

The present study aims to (1) analyze the reliability of the *Dita.te*, a written assessment test based on a dictation task with automatic analysis, and (2) establish normative data for the instrument for children in the 3rd to 6th grade.

## 2. Materials and Methods

### 2.1. Instrument Development

The *Dita.te* is a dictation task associated with an automatic analysis tool constructed using Excel spreadsheets with formulas.

The *Dita.te* tool was designed to integrate all major orthographic components, including stress patterns and prosodic features.

For the selection of targets, phonological, morphological and orthographic criteria were taken into account, based on the triple word form theory, as well as the taxonomy of reading errors, supported by the linguistic criteria proposed by [19], adapted to writing and articulated with the error analysis proposal of [7] and [20]. The following examples of errors are specifically addressed in Table 1.

The current version of *Dita.te* includes 193 words, representing all phonemic segments of European Portuguese (EP) in various word positions and stress patterns, with word length also being controlled as a variable. The number of words is consistent across year of schooling. The text was constructed to be coherent and age-appropriate while containing all target linguistic elements. The frequency of occurrence of all lexical targets included in the instrument was verified using the ESCOLEX database, which is the first lexical database of European Portuguese that provides word frequency statistics based on school textbooks from the 1st to the 6th grade [21]. Almost all (98%) stimuli belong to the lexicon of Portuguese children.

For target selection, 36 lexical items were included from the Crosslinguistic Child Phonology Project—European Portuguese phonology assessment test (CLCP-PE) [22,23], as this is a test constructed considering non-linear phonology; therefore, it phonologically controls for variables such as segment, syllabic structure, position within the word, word stress, and word length. Additional orthographically controlled targets were also selected, with all vowels and diphthongs (oral and nasal) and their orthographic representations, as well as all non-one-to-one correspondences between phonemic and graphic segments.

The text is presented to the child through a dictation task, and the evaluator enters the child’s production results into a spreadsheet designed for this purpose, which allows for automatic counting. The automatic writing analysis tool includes error analysis for consonant segmental substitution (grouped by manner (e.g., <r> for <l>), place (e.g., <s> for <x>), and voicing (e.g., <f> for <v>)), as well as segmental substitution affecting the syllable nucleus (vowels and diphthongs); orthographic substitution (e.g., <x> for <ch>); syllabic form (omissions, insertions, and inversions of graphemes); use of clitics (e.g., omission of <se>); and word boundaries. Additionally, it enables the automatic recording of errors related to graphic word accentuation, punctuation, and the use of uppercase/lowercase letters. The tool also provides error analysis by grammatical class, word length (number of syllables), stress, position within the word, and syllabic structure. Four automatically filled-in sheets are available, properly formatted, to enable a neat and organized printout of the most relevant writing analysis results.

The pilot study [24] mentions the content validity associated with the linguistic criteria used to select the stimuli, guaranteeing the relevance of the phonological, morphological and orthographic control associated with the stimuli used in the test. These criteria are reflected in the spreadsheet and allow the user to quickly analyze (qualitatively and quantitatively) the child’s writing performance. Eighty participants were included in the pilot study. Based on the data analysis of the pilot study, a number of changes were introduced: the grapheme inventory and its corresponding phonemic representations were added, and the number of omitted syllables or words, as well as the number of substituted words, was included. Additionally, the instructions were revised to enhance clarity.

### 2.2. Participants

Three hundred and fifteen children were included in this study. None of the children had any condition (e.g., intellectual disability, hearing impairment, specific learning disorder). European Portuguese was the native language of all children.

Ethical approval was given by an independent Ethics Committee (reference number 4_CEI2022), and informed consent was collected from all carers before any data collection.

Among the 315 children, more were female (*n* = 160, 50.8%). Table 2 presents the sample by gender, age and year of schooling. The minimum age of the total sample was 8 years and 1 month and the maximum age was 13 years and 11 months.

Considering the year of schooling, 62 were in the 3rd grade, 63 were in the 4th, 74 were in the 5th grade, and 116 were in the 6th grade.

### 2.3. Data Collecztion

All data collection sessions were conducted in school classrooms during regular school hours by trained teachers following a protocol. The inclusion criteria for teachers of the 5th and 6th grade was be a teacher teaching the Portuguese course and that for the 3rd and 4th grade was to be the main teacher. The exclusion criteria for teachers of all years of schooling was having a native language other than European Portuguese. Once the schools had given their permission, the data collection began. Before data collection, the research team provided brief training to classroom teachers on the standardized dictation procedure. A written protocol (specific instructions for teachers) was provided (see Appendix A). During the meeting, teachers were allowed to rehearse the dictation procedure, receiving feedback from the research team.

During the task, the teacher read slowly, using short blocks of words to support information retention. No assistance that could affect the writing performance was given. Words were repeated once if requested by a student, and all punctuation marks were clearly indicated. The teacher recorded the time taken to complete the task. After collection, all written samples were independently scored by two trained raters using the *Dita.te* automatic analysis tool to establish inter-rater reliability. The data used was based on a consensus scoring. The principal discrepancy was due to handwriting accuracy, which was discussed. Also, in cases where no consensus could be reached between the two raters, the symbol for “unreadable letter” was used.

### 2.4. Data Analysis

Descriptive statistics were presented as the mean (M), standard deviation (SD), minimum, and maximum values for the variable “number of correctly written words”, considering the entire sample as well as individual school years. Additionally, for the variable “number of correctly written words”, percentiles (P5, P10, P25, P50, P75, P90, and P95) were computed by school year.

Inter-rater reliability was assessed using the Intraclass Correlation Coefficient (ICC).

The reference values for internal consistency proposed by the literature are as follows: very good (when α > 0.900), good (when α is between 0.800 and 0.900), reasonable (when α is between 0.700 and 0.8.0) and weak (when α is between 0.600 and 0.700) [23,24].

An inferential statistical analysis was also conducted to compare the number of correctly written words and the total number of errors by gender and school year. Normality was assessed using the Kolmogorov–Smirnov test, which indicated that the sample did not follow a normal distribution. Consequently, the Mann–Whitney test was used for gender comparisons, while the Kruskal–Wallis test was applied to compare different school years. Post hoc comparisons with Bonferroni correction were also performed to further explore significant differences between school years.

## 3. Results

This section presents the reliability results, as well as the normative data for children from the 3rd to 6th grade, based on descriptive and inferential statistics.

Internal reliability was calculated using Cronbach’s alfa (α = 0.929), indicating a high internal consistency. The correlation between items was also analyzed. The ICC was performed, and the result was 0.925.

*Descriptive and Inferential Statistics*

The results presented are organized by school year. First, the number of correctly written words was analyzed. The data reveal that the number of correctly written words increases proportionally with the school year, stabilizing in the 4th grade (Table 3). These findings have important implications for both educational and clinical contexts. When a child fails to meet the expected performance criteria, referral to specialized support services is recommended.

The percentiles for the number of correctly written words were also calculated by school year (Table 4).

Table 5 presents the data, by school year, regarding the mean, standard deviation, minimum, maximum, and percentiles for the total number of errors, as well as grouped errors, including segmental substitution errors that differ in manner, place, and voicing, errors affecting the syllable nucleus, orthographic substitution errors, and omission, insertion, and inversion errors.

The data reveal that the number of errors decreases as the school year progresses, with errors affecting the syllable nucleus being the most frequent across all school years (e.g., substitution of vowels with non-bidirectional correspondence of letter–sound, such as <o> by <u>, and simplification of syllabic structure with glide omission and written form of nasal diphthongs, which involves phonological, morphological and orthographic processing). These are followed by orthographic substitution errors (e.g., orthographic correspondence of phoneme [s] and multiple sounds for one grapheme <x>) and grapheme omission (e.g., omission of grapheme <s> that represents the final coda, due to difficulties in processing a complex syllabic structure). Segmental errors for encoding segments that differ in manner, place, and voicing occur less frequently.

**Figure 2:**
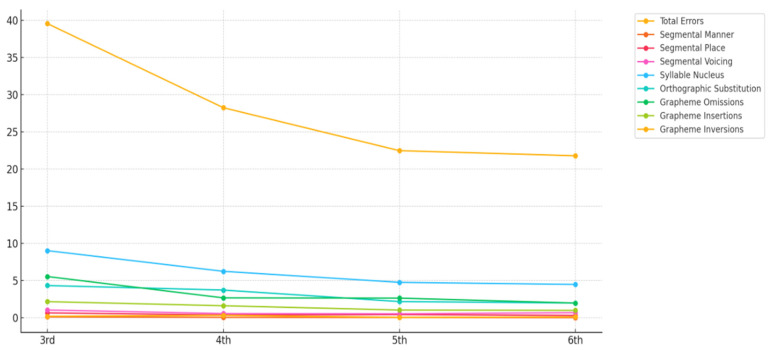
Number of errors (absolute frequency) on *Dita.te*, across years of schooling.

Nevertheless, voicing is the feature that results in the highest number of errors (e.g., substitution of <v> by <f>). The data show that phonological substitution errors regarding consonants are not expected in any school year included in the study (Figure 2).

Inferential statistical analyses were conducted to compare the number of correctly written words and the total number of errors by gender and school year. The possible influence of gender on writing performance was analyzed considering that previous studies have found that girls usually have better writing skills than boys [25,26], although the mechanism underlying gender gaps is not fully understood [27].

The Mann–Whitney test revealed a significant difference between genders for both variables. For the total sample, the median rank for correctly written words was higher for females (U = 9275.500, Z = −3.868, *p* < 0.001), whereas the median rank for the total number of errors was higher for males (U = 9168.500, Z = −4.000, *p* < 0.001). Analyzing the data by years of schooling, a significant gender difference was found in the 6th grade for both variables (U = 1075.0, Z = −3.076, *p* = 0.002, considering the number of correctly written words; U = 1070.0, Z = −3.103, *p* = 0.002, regarding the total number of errors).

Regarding school year comparisons, the Kruskal–Wallis test indicated significant differences across different school years in both the number of correctly written words (H(3) = 28.741, *p* < 0.001) and the total number of errors (H(3) = 27.955, *p* < 0.001).

The post hoc pairwise comparisons for the number of correctly written words revealed significant differences between the 3rd and 4th grades (*p* = 0.003, adjusted for multiple comparisons), the 3rd and 5th grades (*p* < 0.001), and the 3rd and 6th grades (*p* < 0.001). No statistically significant differences were observed between the 4th, 5th, and 6th years.

Regarding the total number of errors, the analysis indicated significant differences between the 3rd and 4th grades (*p* = 0.010), the 3rd and 5th grades (*p* < 0.001), and the 3rd and 6th grades (*p* < 0.001). However, no significant differences were found among the 4th, 5th, and 6th years. These findings suggest that students in the 3rd grade exhibited a significantly lower performance in terms of correctly written words and a higher number of errors compared to students in the subsequent years.

## 4. Discussion

This study aimed to assess the reliability of the *Dita.te*, a written assessment test based on a dictation task with automatic spreadsheet analysis, and establish normative data for text dictation tasks for children from the 3rd to 6th grade in European Portuguese.

### 4.1. Reliability of Dita.te

The *Dita.te* instrument demonstrates strong psychometric properties, as evidenced by its excellent inter-rater reliability (ICC = 0.925) and high internal consistency (Cronbach’s α = 0.929). These values exceed widely accepted benchmarks [28,29], underscoring the tool’s capacity to deliver consistent outcomes regardless of the individual inputting the data. Such consistency is particularly important in both research and clinical contexts, where dependable assessment is vital for accurately identifying children who may need additional support in their writing development.

These high reliability scores likely stem from the instrument’s systematic development, which was informed by established linguistic theory and involved the careful control of key linguistic variables. Furthermore, the automated scoring system enhances reliability by minimizing the risk of human error in both scoring and error categorization, and could reduce the diagnostic time in speech–language pathology. These results are consistent with previous research highlighting the advantages of computerized tools in the assessment of language and literacy skills [17,18].

Girls performed better than boys in the 6th grade. This impact of gender on the results was also confirmed in [25].

The findings provide strong evidence for the reliability of the instrument and offer insights into the developmental progression of writing skills in this population.

### 4.2. Developmental Patterns in Orthographic Skills

The data obtained using *Dita.te* shows that the number of correctly written words increases according to the grade level, as observed in [4,9,30]. This progression reflects the expected developmental trajectory as children gain more exposure to written language and formal instruction in spelling and writing.

A notable finding is the significant development between the 3rd and 4th grades, followed by relative stabilization in later grades. This pattern suggests that orthographic skills in European Portuguese undergo substantial development during the early elementary years, with a plateau reached around 4th grade. This suggests that there should be a focus on writing skills in the early stages of learning. This finding aligns with research in other alphabetic languages suggesting that basic orthographic competence is largely established by middle elementary school [30], though refinement continues in later years.

As revealed by [30], there was a consistent decrease in the average proportion of misspelt words and the total number of errors across grade levels.

The results obtained align with [30], in which phonographic errors were the type of error with the lowest occurrence rate at all grade levels and tasks. Similarly, in [4], with a word dictation task, there was a consistent decrease in phonological errors (statistically significant) and a stable proportion of spelling and morphological errors from the 1st to the 5th grade.

The reduction in errors across grade levels was not uniform across error types. Errors affecting the syllable nucleus (vowels and diphthongs) remained the most prevalent across all grade levels, which is consistent with other European Portuguese studies [31]. The persistent challenge of representing vowels accurately likely reflects the complex phoneme–grapheme relationships in European Portuguese vowel systems, where multiple orthographic representations exist for similar phonological units. So, it is important to provide strategies to address vowel-related difficulties (e.g., phoneme–grapheme mapping drills, visual cues).

As shown in Figure 2, phonological errors related to consonant features (manner, place, and voicing) occur infrequently across all grade levels, because these basic sound–letter correspondences are mastered early in literacy development. This pattern supports previous findings that phonological skills related to consonant representation tend to stabilize earlier than other orthographic skills [7,24]. This error type typically occurs in early writing acquisition during school [7] and in children with developmental language and literacy disorders [6], and does not occur or occurs in a residual way after the 3rd grade.

Errors (substitutions and omissions) that affect the writing of non-biunivocal relationships between sounds and letters (spelling errors) also show slightly higher rates than the others.

Phonological errors, apart from those related to the representation of the syllabic nucleus, tend to resolve earlier than other types of errors. Nonetheless, a residual presence of other error types may still be observed. This pattern indicates that between the 3rd and 6th years of schooling, there is a general stabilization of orthographic competence. Error rates beyond this developmental window may serve as potential indicators of underlying language or learning disorders [24,32].

Our findings also provide support for the Triple Word Form Theory [5,11] in European Portuguese orthographic development. The pattern of errors observed across grade levels shows that children gradually develop integrated representations across phonological, orthographic, and morphological domains. From the 3rd grade onwards, children are moving from phonological-based cues to using morphological and orthographic strategies. Morphological knowledge is especially important when learning to write more complex word forms (e.g., irregular verbal inflexion) and even unfamiliar words. It allows the activation of phonological and orthographic knowledge, which improves the correctness of writing [33]. Morphological awareness could be fostered in classrooms to support orthographic development (e.g., explicit instruction on morphemes, like teach common prefixes, suffixes, and roots).

The correlation analysis further supports this interactive model, showing that different error types are interconnected rather than representing entirely separate modular processes.

The persistent challenges with vowel representation (syllable nucleus errors) highlight the unique aspects of European Portuguese orthography, where the semi-transparent nature of the writing system [14] creates particular challenges for learners.

### 4.3. Clinical and Educational Implications

The *Dita.te* instrument offers several significant advantages for educational and clinical settings.

#### 4.3.1. Clinical Utility

The automatic analysis feature substantially reduces the time required for error analysis, allowing for more efficient assessment procedures. This efficiency could promote the more frequent monitoring of writing development, enabling the earlier identification of children who may require additional support.

The comprehensive error classification system provides detailed insights into specific areas of difficulty, which could inform targeted intervention approaches.

The results obtained in this study reinforce the importance of conducting an error analysis that considers the assumptions of the Triple Word Form Theory, as this analysis allows for the specification of errors, identifying their genesis, which is crucial for establishing appropriate interventions. On the other hand, the normative data obtained in the present study provide insights into children’s writing performance during these years of schooling, as there had been no data until now.

#### 4.3.2. Pedagogical Benefits

The established norms for different grade levels provide valuable reference points for identifying children whose writing development deviates from age-expected patterns. This normative data could enhance the early detection of writing difficulties, potentially leading to more timely interventions.

For instance, the finding that syllable nucleus errors persist across grade levels suggests that this area may benefit from focused instruction even in later elementary grades.

Specifically, it will be important to disseminate specific programs that include strategies regarding phonological knowledge that can be used in educational contexts. For European Portuguese, the “Ilha Periscópio” is an open-access, validated educational resource provided by the Portuguese Ministry of Education, which includes important activities for developing writing skills: e.g., an activity to promote the relation between sound and its written representation—the letter—in isolation, in consonant–vowel combinations, or within the context of the whole word; the phonological patterns associated with plural formation in words with the nasal diphthong [ɐ̃$\tilde{w}$] spelled as <-ão>; and the automation of the relationship between the nasal diphthong in third person plural verb forms and its written representation [34].

## 5. Conclusions

The *Dita.te* instrument demonstrates excellent reliability and provides valuable normative data for assessing writing development in European Portuguese-speaking children from the 3rd to 6th grade. With the application of *Dita.te* to 315 children from the 3rd to the 6th grade, it was possible to conclude that the number of correct words progressively increased, with the most notable difference occurring in the transition from the 3rd to the 4th grade, followed by relative stabilization; this indicates that the 3rd to 4th-grade transition may be a critical window for focused spelling instruction. The error patterns observed across grade levels support the theoretical framework of the Triple Word Form Theory, indicating that children progressively integrate phonological, orthographic, and morphological knowledge as they develop writing proficiency. The persistent challenges with vowel representation highlight the unique features of European Portuguese orthography and suggest potential targets for focused instruction.

In the future, we will aim to analyze the other variables available in *Dita.te*, expand the sample to other geographical regions, and examine the tool’s performance with clinical populations. Additionally, the development of a word dictation version for younger children (e.g., 2nd grade) would extend the tool’s utility to earlier stages of writing development. Also, the authors intend to develop an app or web-based application of *Dita.te* that is more user-friendly. Given that *Dita.te* is a writing assessment tool based on text dictation, an app would allow for more efficient data collection, automated scoring, the real-time analysis of students’ writing skills, and the automatic final report of errors. It would make the tool more accessible to educators and clinicians, supporting the immediate identification of writing difficulties in educational settings.

The automatic analysis feature of the *Dita.te* offers practical advantages for educational and clinical settings, enabling efficient assessment and detailed error analysis. These features, combined with the established normative data, make the *Dita.te* a valuable tool for monitoring writing development. This tool will be crucial to identify early signs of writing difficulties in children who may benefit from additional support.

## Figures and Tables

**Table 1:** Examples of errors of *Dita.te*.

Linguistic Criteria	Examples
Word grammatical class	Nouns, verbs, verbs + Pronouns, adjectives, adverbs, conjunctions, numerical quantifiers, pronouns, determiners, prepositions, contractions (preposition + article), interjection
Distinctive features	Voicing, place, manner
Syllabic structure	V, V(N), VG, VG(N), VGC, VC, V(N)C, CV, CV(N), CVG/CGV, CVG(N), CVC, CVGC, CVG(N)C, CV(N)C, CCV, CCV(N), CCVC, CCVG
Word stress	Strong, weak
Position within the word	Initial, medial, final
Word length	Monosyllables, disyllables, trisyllables, polysyllables
Morphological criteria	Errors with clitic pronouns and word boundary errors

**Table 2:** Distribution of the sample by gender, age and year of schooling.

Year of Schooling	Female (%)	Male (%)	Mean Age ± DP
3rd	23 (37.1%)	39 (62.9%)	8.35 ± 0.52
4th	26 (41.3%)	37 (58.7%)	9.27 ± 0.45
5th	42 (56.8%)	32 (43.2%)	10.46 ± 0.50
6th	69 (59.5%)	47 (40.5%)	11.31 ± 0.50
Total	160 (50.8%)	155 (49.2%)	-

**Table 3:** Number of correctly written words by school years.

School Year	Mean	SD	Minimum	Maximum	Mean Difference (vs. Previous Grade)
3rd grade (n = 62)	165.05	16.289	123	191	-
4th grade (n= 63)	172.79	16.205	129	192	7.74
5th grade (n = 74)	176.32	11.568	136	191	3.53
6th grade (n = 116)	176.76	10.597	138	192	0.44

Legend: SD—standard deviation.

**Table 4:** Percentiles for the number of correctly written words by school years.

Percentiles	Number of Correctly Written Words			
	3rd Grade	4th Grade	5th Grade	6th Grade
5	131.30	132.00	154.00	155.70
10	136.60	141.80	158.00	162.70
25	158.00	167.00	171.00	172.00
50	166.50	177.00	179.50	177.50
75	176.00	186.00	184.00	185.00
90	185.70	188.60	188.00	189.00
95	190.85	189.80	188.25	190.00

**Table 5:** Errors by school years.

School Year	Errors	Mean	SD	Min.	Max.	P25	950	P75
**3rd grade** (n = 62)	Total number of errors	39.55	26.388	2	105	19.50	36.00	54.00
	Segmental substitution errors by manner	0.13	0.338	0	1	0.00	0.00	0.00
	Segmental substitution errors by place	0.66	1.159	0	5	0.00	0.00	1.00
	Segmental substitution errors by voicing	1.05	1.530	0	6	0.00	0.50	1.00
	**Errors affecting the syllable nucleus**	**9.03**	**8.049**	**0**	**36**	**3.75**	**6.50**	**13.00**
	**Orthographic substitution errors**	**4.34**	**3.715**	**0**	**13**	**1.00**	**4.00**	**7.00**
	**Grapheme omissions**	**5.55**	**7.130**	**0**	**33**	**1.00**	**3.00**	**7.25**
	**Grapheme insertions**	**2.18**	**2.532**	**0**	**10**	**0.00**	**1.00**	**3.00**
	Grapheme inversions	0.21	0.577	0	3	0.00	0.00	0.00
**4th grade** (n = 63)	Total number of errors	28.24	25.193	1	112	10.00	22.00	33.00
	Segmental substitution errors by manner	0.06	0.304	0	2	0.00	0.00	0.00
	Segmental substitution errors by place	0.38	0.728	0	3	0.00	0.00	1.00
	Segmental substitution errors by voicing	0.57	0.875	0	3	0.00	0.00	1.00
	**Errors affecting the syllable nucleus**	**6.25**	**7.128**	**0**	**29**	**2.00**	**4.00**	**7.00**
	**Orthographic substitution errors**	**3.73**	**4.502**	**0**	**23**	**1.00**	**2.00**	**5.00**
	**Grapheme omissions**	**2.68**	**3.325**	**0**	**16**	**0.00**	**2.00**	**4.00**
	Grapheme insertions	1.62	2.648	0	13	0.00	1.00	2.00
	Grapheme inversions	0.33	1.032	0	5	0.00	0.00	0.00
**5th grade** (N = 74)	Total number of errors	22.47	18.218	1	89	10.75	17.00	28.25
	Segmental substitution errors by manner	0.05	0.228	0	1	0.00	0.00	0.00
	Segmental substitution errors by place	0.43	0.812	0	4	0.00	0.00	1.00
	Segmental substitution errors by voicing	0.54	0.814	0	4	0.00	0.00	1.00
	**Errors affecting the syllable nucleus**	**4.77**	**4.153**	**0**	**20**	**2.00**	**3.00**	**6.00**
	**Orthographic substitution errors**	**2.19**	**2.065**	**0**	**9**	**1.00**	**2.00**	**3.00**
	**Grapheme omissions**	**2.65**	**4.305**	**0**	**27**	**0.00**	**1.00**	**3.00**
	Grapheme insertions	1.05	1.507	0	8	0.00	1.00	1.25
	Grapheme inversions	0.07	0.253	0	1	0.00	0.00	0.00
**6th grade** (n = 116)	Total number of errors	21.78	19.023	1	148	10.00	18.00	28.75
	Segmental substitution errors by manner	0.03	0.159	0	1	0.00	0.00	0.00
	Segmental substitution errors by place	0.32	0.537	0	2	0.00	0.00	1.00
	Segmental substitution errors by voicing	0.69	1.075	0	6	0.00	0.00	1.00
	**Errors affecting the syllable nucleus**	**4.49**	**3.722**	**0**	**24**	**2.00**	**3.50**	**6.00**
	Orthographic substitution errors	1.99	1.931	0	9	0.25	1.50	3.00
	Grapheme omissions	1.99	2.462	0	15	0.00	1.00	3.00
	Grapheme insertions	1.00	1.389	0	6	0.00	0.00	2.00
	Grapheme inversions	0.13	0.428	0	3	0.00	0.00	0.00

Legend: SD—standard deviation; Min.—Minimum; Max.—Maximum. The most frequent error types are in bold.

## Data Availability

The datasets generated during and/or analyzed during the current study are available from the corresponding author upon reasonable request.

## Appendix A

The instructions for teachers

1. The teacher should dictate to the class and record the time taken.
2. Privacy dividers should be placed between each student to prevent copying.
3. The children should write on standardized lined paper provided by the research team.
4. Read, slowly, two or three words at a time or small blocks of information that require you to retain the information (small sentence).
5. Do not provide any kind of help that compromises the results obtained.
6. Repeat words when asked by a student (once).
7. At the top of the sheet, write the student’s initials, grade, class, gender, and date of birth.
8. Provide positive reinforcement when necessary, for example: “Very good, we’re almost there…”, “Nice work!”.
